# Dicentric (7;12)(p11;p11) in T/Myeloid Mixed-Phenotype Acute Leukemia

**DOI:** 10.4274/tjh.galenos.2021.2021.0280

**Published:** 2021-08-25

**Authors:** Smeeta Gajendra, Akshay Ramesh Gore, Nitin Sood, Manorama Bhargava

**Affiliations:** 1 Department of Laboratory Oncology, All India Institute of Medical Sciences, Dr. B.R.A. Institute Rotary Cancer Hospital, New Delhi, India; 2 Department of Hematopathology, Medanta - The Medicity, Gurgaon, India; 3 Department of Medical Oncology & Hematology, Medanta - The Medicity, Gurgaon, India

**Keywords:** Dicentric (7;12), Mixed-phenotype acute leukemia, Fluorescence in situ hybridization, ETV6/RUNX1

## To the Editor,

Mixed-phenotype acute leukemia (MPAL) is a rare heterogeneous group of acute leukemias with immunophenotypic co-expression of more than one cell lineage, which could be bilineal or biphenotypic. MPAL could be further classified as B/myeloid, T/myeloid, B/T-lymphoid, and, more rarely, trilineage B/T/myeloid. “MPAL, T/myeloid, not otherwise specified” is a rare variant of this disease accounting for <1% of all leukemias [1]. It is associated with male predominance, frequent lymphadenopathy, and poor prognosis [2]. Most of the cases have clonal chromosomal abnormalities, but none are specific for this group. Here, we report a case of T/myeloid bilineage acute leukemia with unusual cytogenetic features upon karyotyping and fluorescence in situ hybridization (FISH), with karyotyping showing a dicentric chromosome between the derivative chromosome 7 and chromosome 12, dic(7;12)(p11;p11).

**Figure 1:**
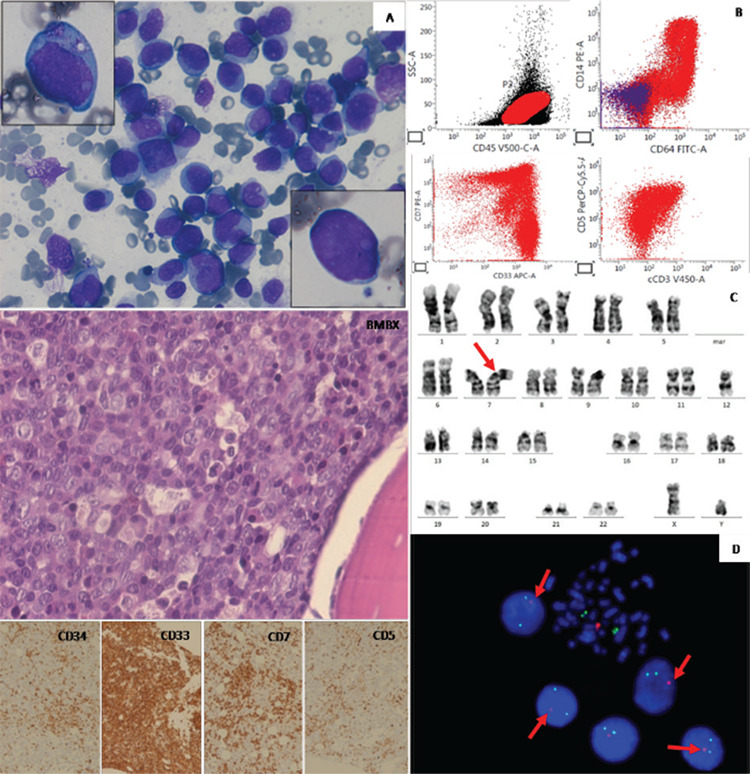
A) Bone marrow aspirate showing two distinct populations of blasts (insets showing two types of blasts). Bone marrow biopsy (hematoxylin and eosin stain; 400^x^) with immunohistochemistry showing two populations of blasts with mixed myeloid (CD33) and T lymphoid markers (CD7 and CD5). B) Flowcytometry showing two blast populations positive for myeloid CD33, CD64, CD14, and T lymphoid markers (cCD3, CD5, CD7). C) Karyogram showing dicentric chromosome formed between 7 and 12, dic(7;12)(p11;p11), with GTG staining and banding method, 100^x^ oil immersion, Carl Zeiss Axioscope Z2, processed using IKAROS software. D) Fluorescence in situ hybridization analysis with interphase nuclei and metaphase showing deletion of the *ETV6 (TEL)* gene localized on 12p 13.2 using the *ETV6/RUNX1* DC, DF probe (*ETV6* - orange, *RUNX1* - green; ZytoVision, Germany).

A 51-year-old male presented with generalized weakness and loss of appetite. On examination, he had pallor and abdominal mass with hepatomegaly (2 cm below the right costal margin). Abdominal computed tomography showed multiple large lymph nodes in the paraaortic, aortocaval, paracaval, celiac axis, periportal, and mesenteric regions, the largest measuring 3.0x2.5 cm in the left paraaortic region. Fine-needle aspiration of the paraaortic lymph node showed clusters of pleomorphic cells, suggesting a lymphoproliferative lesion. Complete blood count showed hemoglobin of 7.7 g/dL, total leukocyte counts of 9.0x10^9^/L, and platelets of 156x10^9^/L. Peripheral blood smear and bone marrow aspirate showed 70% and 85% blasts, respectively. Morphologically, there were two distinct populations of blasts, one that was larger with 2-3 prominent nucleoli and moderate to abundant amounts of granular cytoplasm (Figure 1A), while the other was of medium size with inconspicuous nucleoli and scanty agranular cytoplasm (Figure 1B). Upon flowcytometric immunophenotyping (Figure 1E), there were two different blast populations seen in the CD45-moderate blast region, extending into the monocytic region: a blast population in the CD45-moderate region with high side scatter (~60% of blasts), extending to the monocytic region, was positive for cMPO, CD13, CD33, CD64, CD14, HLA-DR, CD38, CD11b, CD71, CD123, CD56, CD4, CD117, CD7, and CD11c and negative for cCD79a, CD19, CD10, cCD3, CD8, CD3, CD5, CD2, CD1a, and CD16, while the blast population present in the CD45-moderate region with low side scatter (~40% of blasts) was positive for cCD3, CD3, CD7, CD5, CD34, HLA-DR, CD38, CD99, TdT, and CD33 and negative for cCD79a, cMPO, CD19, CD10, CD1a, CD2, CD4, CD8, CD13, CD14, and CD64. Immunohistochemistry based on bone marrow biopsy also showed sheets of blasts comprising two populations of blasts with myeloid (CD33, CD117) and T lymphoid markers (CD3, CD5, CD7). The overall features were consistent with MPAL of myeloid and T lymphoblastic lineage (MPAL: T/myeloid). Karyotyping (Figure 1C) showed a dicentric chromosome formed between chromosome 7 and 12: 45,XY, dic(7;12)(p11;p11). Dicentric chromosomes involving 12p are associated with loss of 12p material, often including the *ETV6 (TEL)* gene localized in 12p 13.2. The karyotyping findings were supported by FISH using the ETV6/RUNX1 probe (Figure 1D), which showed deletion of the *ETV6 (TEL) *gene localized on 12p13.2. Real-time quantitative polymerase chain reaction results for *PML-RARA, AML1-ETO, RUNX1:RUNX1T1, CBFB - MYH11, FLT3 ITD* and *TKD, D835, NPM1, BCR-ABL1,* and *KIT* were negative. The patient began hyper-CVAD induction chemotherapy comprising cyclophosphamide, vincristine, doxorubicin, and dexamethasone. On day 19, he developed febrile neutropenia. Blood culture showed growth of *Pseudomonas*, for which he was treated, and he recovered. The patient was assessed after completion of four cycles and he achieved a complete morphological remission with this regimen.

T/myeloid MPAL is rare and characterized by the presence of both T and myeloid lineage markers in immunophenotyping. Although the specific type and frequency of genetic abnormalities associated with T/myeloid MPAL are largely unknown, some chromosomal abnormalities described in the literature commonly include recurrent monosomies 7p and/or 12p [2,3,4,5]. Structural abnormalities in the short arm of chromosome 12 are observed in a broad spectrum of hematological malignancies including myeloid malignancies and acute lymphoblastic leukemia. Various aberrations result in abnormal 12p, including balanced translocations, deletions, and formation of dicentric chromosomes. Dicentric chromosomal abnormalities have been reported in many hematological malignancies including myelodysplastic syndrome, acute myeloid leukemia [6], and acute lymphoblastic leukemia [7,8,9,10], and dic(7;12) is a rare but recurrent chromosomal abnormality described mainly in childhood acute lymphoblastic leukemia [7]. A case of dic(7;12)(p11;p11) in T/myeloid biphenotypic acute leukemia has also been reported; that patient was successfully treated with myeloablative stem cell transplantation [2]. A rare case of new dic(7;12)(p12.21;p12.2) chromosome aberration was also reported in a patient with acute myeloid leukemia with FAB-M1 morphology. It is known that dic(7;12) results in partial monosomies of 7p and 12p, leading to concomitant deletions of tumor suppressor genes from both chromosomes, which plays a role in the pathogenesis of hematological malignancies. As MPAL with dic(7;12) is rarely reported in the literature, the prognostic significance and definite therapeutic regimens for these patients have not yet been established.
